# Prevalence and determinants of work related injuries among small and medium scale industry workers in Bahir Dar Town, north west Ethiopia

**DOI:** 10.1186/s40557-015-0062-3

**Published:** 2015-04-08

**Authors:** Getnet Abebe Molla, Waju Beyene Salgedo, Yohannes Kebede Lemu

**Affiliations:** Department of Internal Medicine, Wollo University, Dessie, Ethiopia; Department of Health Economics, Management and Policy, Jimma University, College of Health Sciences, P.O.Box. 378, Jimma, Ethiopia; Department of Health Promotion and Behavioral Science, Jimma University, College of Health Sciences, P.O.Box. 378, Jimma, Ethiopia

**Keywords:** Occupational health, Work-related injury, Small and Medium industry, Bahr Dar

## Abstract

**Objectives:**

To assess the prevalence and determinants of work-related injuries among small and medium scale industrial workers in Bahir Dar town, northwest Ethiopia.

**Method:**

Cross sectional comparative study design was used. Purposive sampling method was used to choose the specific Kebele 14 of the study area, for its relatively high number of industries. The study units were stratified into small and medium scale industries. All workers who were available at the time of interview were included in the study. A pre-tested questionnaire was used to collect data. Data was analyzed using SPSS for windows 16.0.

**Result:**

A total of 328 and 655 workers from small and medium-scale industries respectively participated in the study. Seven hundred sixty nine (78.2%) were males. Three hundred thirty six workers (34.2%) reported that they had experienced work-related injuries. Sex, monthly salary, age, work experience and use of personal protective equipment were found to be different in the small and medium industries (P < 0.05).

**Conclusion:**

There was a high prevalence of work related injuries. Sociodemographic, socioeconomic, personal work behavior and the working environment have contributed for the injuries. Work-related injuries are assumed to be preventable with the provision of occupational health programs in workplaces. Thus it is recommended that the owners of industries need to focus on training and installing safer work environment and Further studies with large-scale coverage and prospective study designs are warranted.

## Introduction

Globally, poor occupational health and safety results in 271 million work related injuries, 2 million work-related deaths, and 160 million work-related diseases per year. In developing countries including Ethiopia, the risk of having work-related injury is 10 to 20 times higher than that of developed counties. This is because in developing countries, majority of the workforce is employed in small and medium scale industries that do not meet the minimum standards and guidelines set by the World Health Organization and the International Labor Organization for occupational health, safety and social protection [1].

Hundreds of millions of people throughout the world work under circumstances that foster ill health and/or are unsafe. It is estimated that yearly over 1.1 million people worldwide die of occupational injuries and work-related diseases, a figure roughly equivalent to the global annual number of deaths from malaria. An additional problem to workers in Africa is the high prevalence and incidence of HIV/AIDS [2].

According to another study, globally, work-related injuries continue to pose serious public health problems and are leading cause of death, disability and disease. It is estimated that yearly over 2 million people worldwide die of work-related injuries [3].

The stated heavy burden calls for urgent strengthening of the field of occupational hazard prevention and control in Africa. Safer and healthier work conditions can make an important contribution to poverty alleviation and sustainable development. Efficient application of available knowledge to develop practical solutions to overcome the “knowledge application gap” is more important than generating new theoretical knowledge [4].

So far, only few studies have been done to identify work–related injuries and related occupational health and safety problems among large industrial workers in the country. However, information regarding the present status on the prevalence and factors affecting work-related injury is lacking for small and medium scale industries [1].

Since there is poor Epidemiological information regarding the present status on the prevalence and factors affecting work-related injury among small and medium scale industrial workers, it is crucial to add some knowledge to the prevalence, severity and determinants of work-related injuries among these workers. Thus, the aim of this study is, to assess the prevalence of work-related injury, severity, and determinate factors. The finding of this study will help for the development of preventive polices, effective intervention priorities, and any cost-benefit analysis on occupational health and safety legislation and occupational health services.

## Materials and Methods

This study was conducted in Bahr Dar town, the capital city of Amhara regional state, located 570 kms northwest of Addis Ababa, the capital city of Ethiopia from May 7 to May 12, 2013. It is one of the attractive towns with 20 kebeles(villages) and has high tourist flow. It has 166 small and 20 medium scale industries.

A comparative cross-sectional study design was used to assess the prevalence and determinants of work-related injuries among small and medium scale industrial workers.

Initially purposive sampling method was used to choose the specific Keble 14, for its relatively high number of the industries in the study area. Then, the study units were stratified into small and medium scale industries based on number employees being less than or more than 10 respectively. Finally, employees who are directly engaged machine based work processes in the selected industries irrespective of sex and age were included in the study. All 983 workers available at the time of data collection at the selected industries were included.

With regard to variables of measurement, the dependent variable was work-related injuries while the independent variables were Socio-demographic factors (sex, age, salary), work exposure factors(experience, training, educational status, residence, occupation, use of personal protective device) and work environment factor(unguarded machine, poor electric installation, machinery installation, working hours).

Data was collected by 4 trained data collectors using a pretested questionnaire and checklist with close supervision by a supervisor.

Data was analyzed using SPSS for window version 16 and the result was presented in tables. Chi-square test and adjusted odds ratio were used to measure the association of variables at 95 percent confidence interval and p-value of <0.05. Variable found to be significant at p-value <0.05 in bivariate regression analysis were entered into multivariate regression analysis to determine the association (Table 1).

**Table 1:** **Associations of socio-demographic characteristics of respondents with prevalence of work-related injuries by type of industry, Bahirdar Dar, Northwest Ethiopia, May 2013(N = 983)**

**Variables**	**SSI (n=328) N (%)**		**MSI (n=655) N (%)**		**TotalN (%)**		**Odds ratio (95%CI)**
	**Yes**	**No**	**Yes**	**No**	**Yes**	**No**	
**Sex**							
Female	3(0.9)	14(4.3)	58(8.8)	139(21.2)	61(6.2)	153(15.5)	0.72(0.51-0.99)*
Male	115(35.1)	196(57.9)	160(24.4)	298(45.5)	275(27.9)	494(50.3)	1.00
**Age Group**							
14-29	87(26.5)	161(49.1)	146(22.3)	252(38.4)	233(23.7)	413(42.0)	1.282(0.97-1.70)
30+	31(9.4)	49(14.9)	72(10.9)	185(28.2)	103(10.5)	234(23.8)	1.00
**Monthly salary** (Birr)							
≤200	20(6.1)	33(10.1)	10(1.5)	14(2.1)	30(3.1)	47(4.8)	1.98(1.19-3.23)*
201-600	70(21.3)	80(24.4)	140(21.4)	199(30.4)	210(21.4)	279(28.4)	2.97(1.89-4.67)*
600+	28(8.5)	97(29.5)	68(10.4)	224(34.1)	96(9.7)	321(32.7)	1.00
**Job category**							
Daily laborer	20(6.1)	39(11.9)	26(3.9)	72(10.9)	46(4.7)	111(11.3)	1.67(1.09- 2.55)
Mechanic	25(7.6)	48(14.6)	85(12.9)	96(14.6)	110(11.2)	144(14.6)	1.88(1.17- 3.03)*
Machine operator	45(13.7)	12(3.6)	16(2.4)	107(16.3)	61(6.2)	119(12.1)	1.39(0.92- 2.11)
Welder	26(7.9)	22(6.7)	19(2.9)	29(4.4)	45(4.6)	51(5.2)	2.79(1.69- 4.61)*
**Others**	2(0.6)	89(27.1)	72(10.9)	133(20.3)	74(7.5)	222(22.6)	1.00

Note: SSI = small scale industries; MSI = Medium scale industries.

*indicates significant differences.

Ethical clearance was obtained from college of department of health research and community-based education at college of public health and medical sciences of Jimma University. Cooperation letter was written to respective industries and informed verbal consent was obtained from all study participants during data collection.

The operational definitions for this study were:

- **Medium-scale industry**: any industry that uses power driven machine and more than 10 workers.
- **Small-scale industry**: any industry that uses power driven machine and less than 10 workers.
- **Work related injury**: any injury sustained by a worker in connection with the performance of his/her work.
- **Severity of injury**: characterized by death(fatal), hospitalization more than 24 hours and absence from work for more than three days in the last one year (14, 24).

## Result

### Socio-demographic characteristics of the respondents

A total of 983 (328 and 655 workers from small and medium-scale industries respectively) have participated in the study. Seven hundred sixty nine (78.2%) were males and 214 (21.8%) were females with a sex ratio of 3.6:1. 646 (66%) of the respondents were in the age group of 14–29 years. 162 (49.4%) of the respondents were from small industry and 297 (45.3%) of the respondents from medium-scale industry attended secondary school. Majority of the workers, 241(73.5%) from small scale industry and 401(61.2%) from medium scale industries, had a work experience of less than five years respectively. 339 (51.7%) workers of medium scale industries and 150(45.7%) from small scale industry earn a monthly income of 201–600 Birr. Sex, age, monthly salary and work experience were different for the two groups (p < 0.05) while educational level did not show statistically significant difference (Table 2).

**Table 2:** **Socio-demographic characteristics of the respondents by type of industry, Bahirdar Northwest Ethiopia, May 2013**

**Variables**	**SSI (n=328) N (%)**	**MSI (n=655) N (%)**	**Total (n=983) N (%)**	**P-value**
Sex				
Female	17 (5.2)	197 (30.1)	214 (21.8)	78.07
Male	311 (94.8)	458 (69.9)	769 (78.2)	
Age Group				
14-29	248 (75.6)	398 (60.8)	646 (65.7)	21.86
30-45	55 (16.8)	188 (28.7)	243 (24.7)	
45+	25 (7.6)	69 (10.5)	94 (9.6)	
Educational level				
Illiterate	26 (7.9)	41 (6.3)	67 (6.8)	5.53
Can read and write	14 (4.3)	33 (5.0)	47 (4.8)	
Primary	71 (21.6)	183 (27.9)	254 (25.8)	
Secondary	162 (49.4)	297 (45.3)	459 (46.7)	
12+	55 (16.8)	101 (15.4)	156 (15.9)	
Monthly salary (Birr)				
≤200	53 (16.2)	24 (3.7)	77 (7.8)	47.31
201-600	150 (45.7)	339 (51.7)	489 (49.7)	
600+	125 (38.1)	292 (44.6)	417 (42.5)	
Work experience (yrs)				
**≤5**	241 (73.5)	401 (61.2)	642 (65.3)	13.95
**6+**	87 (26.5)	254 (38.8)	341 (34.7)	

Note: SSI=small scale industries; MSI=Medium scale industries.

### Characteristics of work-related injuries

336 (34.2%) of the workers reported that they had experienced some sort of work-related injury in the previous one year. Out of these, 118(36.0%) and 218 (33.3%) occurred in small and medium-scale industrial workers, respectively. Most of the respondents, 201(59.8%), had sustained work related injuries once during the last 12 months. Fingers were the most affected parts of the body with the prevalence rate of 56 (47.4%) in small scale and 74(33.9%) in medium scale industries respectively over all accounting 130 (38.7%) of the total injuries. In terms of type of injury, abrasions were the leading once with a total contribution of 143 (42.6%) (SSI: 51 (43.2%), MSI: 92 (42.2%) (Table 3).

**Table 3:** **Distribution of work-related injury in the last 12 months among respondents by type of industry, Bahir dar town, North West Ethiopia, May, 2013**

**Variables**	**SSI (n=32) N (%)**	**MSI (n=655) N (%)**	**Total (n=98) N (%)**	**P-value**
Work-related injuries				
Yes	118 (36.0)	218 (33.3)	336 (34.2)	0.59
No	210 (64.0)	437 (66.7)	647 (65.8)	
Number of occurrence				
Once	74 (62.7)	127 (58.3)	201 (59.8)	0.46
More than once	44 (37.3)	91 (41.7)	135 (40.2)	
Number of occurrences by part affected				
Finger	56 (47.4)	74 (33.9)	130 (38.7)	0.751
Hand	27 (22.9)	50 (22.1)	77 (22.3)	
Leg	15 (11.0)	39 (16.5)	54 (14.6)	
Eye	8 (3.4)	26(9.6)	34 (7.4)	
Multisite	12 (10.2)	29 (13.3)	41 (12.2)	
Number of occurrences by type of injury				
Abrasion	51 (43.2)	92 (42.2)	143 (42.6)	0.95
Cuts	29 (24.6)	49 (22.5)	78 (23.2)	
Burns	18 (15.3)	31 (14.2)	49 (14.5)	
Fracture	3 (2.5)	8 (3.7)	11 (3.3)	
Others	17 (14.4)	38 (17.4)	55 (16.4)	
Number of occurrences by cause of injury				
Machine	32 (27.1)	67 (30.7)	99 (29.5)	
Electricity	25 (21.2)	72 (33.1)	97 (28.9)	
Hand tools	13 (11.0)	26 (11.9)	39 (11.6)	
Fire and explosion	6 (5.1)	8 (3.7)	14 (4.2)	
Acid and hot substances	4 (3.4)	4 (1.8)	8 (2.3)	
Others	35 (29.7)	40 (18.3)	75 (22.3)	

Note: SSI=small scale industries; MSI=Medium scale industries.

### Work environment and behavioral characteristics of respondents by type of industry

Majority of worker from small-scale industry, 269 (82.0%), work for less than 48 hours and 439 (67.0%) of workers from medium-scale industry reported that they worked more than 48 hours per week. Most of the workers, 553 (56.3%), in both industries did not take Health and safety training. Majorities of workers in both scales use protective devices. Mechanic employers experience more work related injuries than other job categories; accounting 73 (22.3%) in small and 181(27.6%) medium scale industries. Hours worked per week, use of personal protective equipment and job categories are different in the two scales (p < 0.05) but health and safety training didn’t reach a significance level (p > 0.05) (Table 4).

**Table 4:** **Reported work environment and behavioral characteristics of respondents, in Bahir Dar town, Northwest Ethiopia, May 2013**

**Variables**	**SSI (n=328) N (%)**	**MSI (n=655) N (%)**	**Total N (%)**	**X** ^**2**^
Hours worked per week				
≤48	269 (82.0)	216 (33.0)	485 (49.3)	208.08*
48+	59 (18.0)	439 (67.0)	498 (50.7)	
Health and safety training				
Yes	157 (47.9)	273 (41.7)	430 (43.7)	3.15
No	171 (52.1)	382 (58.3)	553 (56.3)	
Use PPE				
Yes	247 (75.3)	353 (53.9)	600 (61.0)	41.24*
No	81 (24.7)	302 (46.1)	383 (39.0)	
Job category				
Daily laborer	59 (18.0)	98 (15.0)	157 (16.0)	16.79*
Mechanic	73 (22.3)	181 (27.6)	254 (25.8)	
Machine operator	57 (17.4)	123 (18.8)	180 (18.3)	
Welder	48 (14.6)	48 (7.3)	96 (9.8)	
Others	91 (27.7)	205 (31.3)	296 (30.1)	

Note: SSI = small scale industries; MSI = Medium scale industries; PPE is personal protective equipment *indicates significant differences.

### Severity of work-related injuries

Of the 336 injured respondents, 71 (21%) were hospitalized, 198 (59%) were absent from work for more than 4 days. As a result, more than 594 working days were lost and two deaths were reported (Table 5).

**Table 5:** **Severity of Work related injuries among small and medium scale industrial workers, in Bahir Dar town, Northwest Ethiopia, May 2013**

**Severity of injury**	**SSI (n=118) N(%)**	**MSI(n=218) N (%)**	**Total (n=336) N (%)**	**X** ^**2**^
Fatal( death)	0(0)	2(0.9)	2(0.6)	13.0*
Major/hospitalization	28(23.7)	43(19.7)	71(21)	
Absent from work >3 days	37(31.4%)	161(73.9)	198(59)	
Total	65(55.1)	206 (94.5)	271(80.7)	

*indicates significant differences.

### Determinants of work-related injuries

Socio-demographic, working environment and behavioral determinants in relation to injury were analyzed by bivariate analysis. Sex, monthly salary and job category were identified as the major socio-demographic determinants of work-related injury for both industrial workers. Male workers, mechanics and welders job holders and workers who get less monthly salary (less than 600 birr) were found to show positive effect on injuries (Table 1).

From the workplace variables, hours worked per week and use of personal protective mechanisms showed significant association with the prevalence of work-related injuries. Engagement in less than or equal to 48 working hours per week and use of personal protective devices are found to be protective factors for the occurrence of injury with the corresponding industries. Although not significant Healthy and safety training in the workplace was protective (Table 6).

**Table 6:** **Associations of environmental factors and behavioral characteristics of respondents with prevalence of work related injuries by type of industries, in Bahir Dar town, Northwest Ethiopia, May 2013 (n = 983)**

**Variables**	**SSI (n=328) N (%)**		**MSI (n=655) N (%)**		**Total (n=983) N (%)**		**Odds ratio (95% CI)**
	**Yes**	**No**	**Yes**	**No**	**Yes**	**No**	
**Hours worked per week**							
≤48	90(27.4)	179(54.5)	38(5.8)	178(27.1)	128(13))	357(36.3)	0.50(0.38- 0.65)*
48+	28(8.5)	31(9.4)	180(27.5)	259(39.5)	208(21.1)	290(29.5)	1.00
**Health and safety training**							
Yes	50(15.2)	107(32.6)	95(14.5)	178(27.1)	145(14.7)	285(28.9)	0.96(0.74-1.26)
No	68(20.7)	103(15.7)	123(18.8)	259(39.5)	191(19.4)	362(36.8)	1.00
**Use PPE**							
Yes	80(24.4)	167(50.9)	115(17.5)	238(36.3)	195(19.8)	405(41.2)	0.53(0.33-0.08)*
No	38(11.6)	43(13.1)	103(15.7)	199(30.4)	141(14.3)	242(24.6)	1.00
**Work experience yrs)**							
≤5	85(25.9)	156(47.6)	146(22.2)	255(38.9)	231(23.4)	411(41.8)	1.26(0.95- 1.67)
6+	33(10.1)	54(16.4)	72(10.9)	182(27.7)	105(10.7)	236(24)	1.00

Note: SSI=small scale industries; MSI=Medium scale industries; PPE is personal protective equipment.

*indicates significant differences.

## Discussion

Our study was aimed to give a baseline data for the study area and similar towns in the country. In our study, the overall annual prevalence rate of work- related injuries were 342 injuries per 1000 exposed workers. This finding is comparable with the study conducted in Gondar in which the work related injuries were 335 injuries per 1000 exposed workers [1]. On the other way, our finding showed markedly greater annual prevalence of work related injury in large-scale in Addis Ababa and other developing countries like Lebanon [5,6]**.** Such variations might be emanated from individual and complex interplay of different situations in the working environments like level of training, use of personal protective devices, and strengths of occupational health services.

Our analysis indicated that statistically significance difference was not observed between small and medium scale industrial workers (P > 0.05). Even though, some studies support this finding [7], it seems in the contrary to the context of developing countries like Ethiopia where there is rapid industrialization, which may result in increased number of small industries and respective high employment unlike medium ones. Majority of workers in these industries were uneducated, unskilled, untrained and inexperienced with the tools and the hazards associated with the work process which can increase the risk of work related injuries [8].Furthermore, there are contrasting and contradicting debates that suggest small-scale industries are more dangerous to work in small industries than that of medium ones [7-9]. Different factors might have contributed for this discrepancy including the recall and response biases, lack of difference in working conditions, working environment and status of workforce in both types of industries.

Although,71 (21%) were hospitalized and 198 (59%) were absent from work for more than 4 days resulting in more than 594 working days lost and two deaths, the severity of work-related injury was not associated with the type of industry. Even though, the severity of injury cannot justified to be associated with size of industry due to its insignificant number of death in this study, costs were inherent characteristics of an increase in severity of injuries which was proved by a Study done in Lebanon that resulted in three (0.1%) deaths which consumed 3.8% of the overall costs. Furthermore, injuries that resulted in the hospitalization were 12 times costly than those did not [9,10].

Sex was found to have a significant difference between the two industries (P < 0.005) where more males were more vulnerable to injuries, in both types of industries. This is may be attributed to the energetic work handling experience of males in the usual practice.

Salary, age group and work experience were found to be different in the small and large industries (P < 0.005), while, unlike the common expectation, level of education is not statistically different in the two industries (P > 0.05).

Majority of the workers had a work experience of less than five years (241(73.5%) and 401 (61.2%) in small scale and medium scale industries respectively. The salary of the workers in both types of industries ranged between 600–2001 Birr. The finding of this study comparable with a study conducted in Gonder [1], which puts the age, sex, salary and work experience as having a statistically significant difference in the two industries, i.e. small and medium, while level of education favors the opposite.

Regarding to the characteristics of work-related injuries, most of the respondents 201(59.8%) had sustained work related injuries. Nevertheless, there is no statistically significant difference between the two industries with respect to frequency of occurrences (P > 0.05). This is contrary to the study done Gonder [4]. Fingers (56 (47.4%) of small scale and 74 (33.9%) medium scale) are usually most commonly affected body parts in both industries, overall accounting 130 (38.7%). In terms of type of injury abrasions are the leading once with a total contribution of 143 (42.6%) (SSI: 51 (43.2%), MSI: 92 (42.2%). Number of occurrences by type of injury, part of body affected and causes of injury are not found to be statistically different in the two industries.

Another important point to discuss is work environment and behavioral characteristics of respondents: majority of small scale (269 (82.0%)) workers work for less than 48 hours and of medium-scale workers (439 (67.0%)) reported that they worked more than 48 hours per week. Hours worked per week was significantly associated with the type of factory (P < 0.05). More hours per week was spent among medium-scale industrial workers and majority of less hours per week are spent for small-scale industries (p < 0.05 (Table 1)).

Most of the workers, 553 (56.3%), in both industries did not take Health and safety training. On the other hand, majorities in both scales use protective devices. Personal handling of protective devises was also significantly associated with the type of factory (p < 0.05). Different studies support this fact (9 10). The multivariate analysis had not indicated that more hours per week was statistically significant among medium- scale industrial workers while Health and safety training and proper handling of personal protective devises were more significant characteristics of small scale industrial workers. The mechanical work employees experienced more work related injuries than any other jobs, accounting 73 (22.3%) in small and 181 (27.6%) medium scale industries. This is also evidenced in other studies [11,12].

## Conclusion

In conclusion, this study revealed that there is high prevalence of work related injuries when compared to some studies in developing countries. Socio-demographic, socio-economic, personal work behavior and the working environment are found to be contributory to the injuries. The type, magnitude and depth of injury are likely to bring significant changes in productivity and affect expenses through medical and compensation costs.

Thus it is recommended that the owners of both small and large-scale industries have to give more attention for improving occupational safety measures and safe working environment through training, routine use of protective devices and promoting healthy and safety working environment to foster health to the valuable workforces. Further studies with large-scale coverage and prospective study designs are warranted.

### Limitation of the study

A recall bias due its short-time and cross-sectional nature.
